# Conditions Associated with Circulating Tumor-Associated Folate Receptor 1 Protein in Healthy Men and Women

**DOI:** 10.1371/journal.pone.0096542

**Published:** 2014-05-08

**Authors:** Linda E. Kelemen, James D. Brenton, Christine Parkinson, Hayley C. Whitaker, Anna M. Piskorz, Ilona Csizmadi, Paula J. Robson

**Affiliations:** 1 Department of Population Health Research, Alberta Health Services, Calgary, Alberta, Canada; 2 Department of Medical Genetics, University of Calgary, Calgary, Alberta, Canada; 3 Cancer Research United Kingdom Cambridge Institute, University of Cambridge, Li Ka Shing Centre, Cambridge, United Kingdom; 4 Department of Oncology, University of Cambridge, Hutchison/Medical Research Council Research Centre, Cambridge, United Kingdom; 5 National Institute for Health Research Cambridge Biomedical Research Centre, Cambridge, United Kingdom; 6 Cambridge Experimental Cancer Medicine Centre, Cambridge, United Kingdom; 7 Departments of Oncology and Community Health Sciences, University of Calgary, Calgary, Alberta, Canada; 8 Alberta Health Services, Edmonton, Alberta, Canada; 9 Department of Agricultural, Food and Nutritional Sciences, University of Alberta, Edmonton, Alberta, Canada; Wayne State University, United States of America

## Abstract

**Background:**

Serum concentrations of the tumor-associated folate receptor 1 (FOLR1) protein may be a marker for early cancer detection, yet concentrations have also been detected in cancer-free women. We investigated the conditions associated with circulating FOLR1 protein in healthy individuals and sought to clarify the range of normal serum values.

**Methods:**

Sera of cancer-free men and women (N = 60) enrolled in a population-based cohort study in Alberta, Canada were analyzed for FOLR1 protein using an electrochemical luminescence immunoassay. Dietary, lifestyle, medical and reproductive history information was collected by questionnaires. Differences in serum FOLR1 concentrations between groups were assessed by non-parametric tests, and predictors of serum FOLR1 concentrations were estimated using multivariable linear regression.

**Results:**

Median serum FOLR1 concentration was higher in women (491 pg/ml, range = 327–693 pg/ml) than in men (404 pg/ml, range = 340–682 pg/ml), P = 0.001. FOLR1 concentration was also positively associated with vitamin A intake (P = 0.02), and showed positive trends with age and with oral contraceptive hormone use among women and an inverse trend with body mass index. All variables examined explained almost half of the variation in serum FOLR1 (model *R^2^* = 0.44, P = 0.04); however, the retention of gender (P = 0.003) and vitamin A intake (P = 0.03) together explained 20% (P = 0.001) of serum FOLR1 variation. No other predictor was significant at P<0.05.

**Conclusions:**

The positive association between serum FOLR1 concentration and female gender independent of an age effect suggests caution against statements to exploit serum FOLR1 for early cancer detection without further understanding the biological underpinnings of these observations. Serum FOLR1 concentrations may be influenced by the steroid retinoic acid (vitamin A) but do not appear to be associated with folate nutritional status. These findings require confirmation in larger independent studies.

## Introduction

Folate receptor 1 (FOLR1) is a membrane-bound protein with high affinity for binding nanomolar concentrations of folate and, in particular, the oxidized form of folate, folic acid [1], [2]. FOLR1 exhibits restricted expression in normal epithelial tissues, including kidney tubules and intestine, placenta and choroid plexus, where it prevents against folate losses from excretion and prioritizes folate uptake by critical tissues, respectively [1]. It is also expressed in specific malignant tumors of epithelial origin [1] and is currently under investigation as a diagnostic and chemotherapeutic target [3]–[8].

FOLR1 expression has been associated with various factors. In non-population-based samples (cell cultures, animal models and anonymous tumor bank samples), negative correlates of FOLR1 expression in tissue or cell lines were 17β-estradiol [9], [10] and folate concentrations [11]–[13] and positive correlates were tamoxifen [9], glucocorticoid receptor [14] and retinoic acid [15]. In a clinical population, however, FOLR1 expression in ovarian tumors was associated positively with multivitamin use [16]. Understanding the relevance of these exposures at a population level is needed. For example, adipose tissue can produce 17β-estradiol, and the increasing prevalence of obesity may influence *in vivo* FOLR1 expression. Exposure to folic acid is also common due to mandatory folic acid fortification of grain products in several countries [17]–[19] and from the popularity of folic-acid containing multivitamin use [20].

A soluble form of the receptor with the folate binding site intact arises from proteolytic cleavage of the glycosylphosphatidylinositol (GPI) membrane anchor by the combined action of a membrane-associated protease and GPI-specific phospholipases C and D, which are abundant in plasma [21]–[23]. The existence of a soluble form was first recognized 40 years ago and referred to as a serum folate binding protein [24]. At that time, investigations focused on correlates of total serum folate binding capacity. Investigators reported increased binding during pregnancy [25], [26] and among users of oral contraceptive hormones [25]–[27] but no association with folate nutritional status [25], [28]. Binding capacity differences between the genders led to the speculation that the serum folate binders may be influenced by sex hormones [27], [28]. These early, initial findings in small numbers of clinical populations are similar to the correlates of membrane-bound FOLR1 expression observed from contemporary *in vitro* and *in vivo* studies.

More recently, FOLR1 protein was reported in the sera of women without cancer [29], [30]. Before serum FOLR1 protein can be considered for investigation as a marker for early cancer detection [29]–[31], it is important to investigate if it is associated with the same factors reported for total serum folate binding capacity in healthy individuals and to clarify the range of normal serum values. Using a sensitive clinical assay, we measured FOLR1 protein in the sera of men and women without cancer who were enrolled in an ongoing population-based cohort study in Alberta, Canada. We also evaluated associations between various dietary, lifestyle, medical and reproductive factors and serum FOLR1 concentrations.

## Materials and Methods

### Study Subjects

The sampling frame was the Tomorrow Project, which is a prospective cohort study established in Alberta, Canada in 2001 with ongoing recruitment to investigate the associations between various lifestyle factors and chronic disease. Briefly, random digit dialing was used to identify eligible men and women between the ages of 35 and 69 years residing in Alberta who had not been diagnosed with cancer other than non-melanoma skin cancer. Details of the study design are described elsewhere [32]. Between 2001 and 2009, participants completed baseline questionnaires, which included the Canadian Diet History Questionnaire [33]. Subsequently, cohort participants were re-contacted and invited to attend a clinic to provide a blood sample, complete a follow-up health and lifestyle questionnaire, and to be measured for height and body weight for calculation of body mass index (BMI in kg/m2). The time lapse between completion of the Diet History Questionnaire at baseline and blood sampling at the follow-up clinic visit was a median of 5.3 years (range: 1.2 to 10.3 years). Most participants (N = 40 [67%]) provided a non-fasting venous blood sample within one week of the clinic visit, another 16 (27%) provided a blood sample up to 6 months following the clinic visit, and another three (two males and one female) up to 1 year and one male at 2 ½ years following the clinic visit. Blood was fractionated into multiple aliquots of 0.5 ml and stored at −80°C. In March 2013, an aliquot was thawed and 25 µl shipped on dry ice to Cambridge, UK for analysis (see below). For the current investigation, we excluded participants who were pregnant or those diagnosed with cancer by cross-checking against the Alberta Cancer Registry.

We identified 60 healthy men and women from those who provided a blood sample using stratified sampling for extreme categories of total folate intake (food folate plus folic acid from food fortification and vitamin supplements) estimated from the baseline Diet History Questionnaire (≤250 µg/d versus ≥600 µg/d), and BMI (BMI≤25 versus BMI≥30) and oral contraceptive use (ever, never: women only) assessed at the time of the clinic visit. We also obtained information on the following variables: age at clinic visit, gender, total vitamin A intake (continuous), alcohol consumption (ever, never), total energy intake (continuous) and current smoking habit (daily, occasionally, none) and for women only: number of pregnancies including live births, still births or miscarriages, lifetime number of months having breast fed and use of postmenopausal hormones (ever, never). These variables were selected to represent hormone-mediated and vitamin exposures that were associated with FOLR1 expression in previous studies [9]–[16].

### Ethics Statement

Informed consent was obtained from all participants recruited into the Tomorrow Project and the Alberta Cancer Research Ethics Committee approved both the cohort and current study protocols.

### Folate Receptor 1 ImmunoAssay

The serum FOLR1 assay was developed as a 2-site immunoassay on the MesoScaleDiscovery (MSD) Sector 6000 Electrochemical luminescence immunoassay system (MesoScaleDiscovery, UK). MSD standard bind plates were coated with 30 µl of a monoclonal anti-human FOLR1 antibody diluted in PBS (mouse anti-FOLR1, R & D Systems, UK), sealed and incubated at 4°C overnight. The plate was then washed 3 times with PBS-Tween MSD wash buffer and blocked with 150 µl MSD Blocker A for a minimum of 1 hour at room temperature. The plate was washed once before commencing the assay.

The FOLR1 standard was reconstituted as recommended by the manufacturer (R & D Systems, UK). To generate a 6-point standard curve, 25 µl of the FOLR1 standard was pipetted into each well with 25 µl DELFIA Diluent II (Perkin Elmer, MA, USA) zero standard to achieve concentrations ranging between 49 and 12,000 pg/ml. For participant samples, 10 µl of sample was pipetted into each well with 10 µl DELFIA Diluent II. Plates were sealed, incubated on a plate shaker at slow speed for two hours at room temperature and washed three times with MSD wash buffer. 25 µl biotinylated goat polyclonal anti-human FOLR1 (R & D Systems, UK) was added at a dilution of 1∶100 in MSD Diluent 100. Plates were sealed, incubated on a plate shaker at slow speed for two hours at room temperature and washed three times with MSD wash buffer. 25 µl MSD streptavidin Sulpho-TAG (MesoScaleDiscovery, UK) was added at a dilution of 1∶1000 in MSD Diluent 100. Plates were sealed, incubated on a plate shaker at slow speed for 30 minutes at room temperature and washed three times with MSD wash buffer. We then added 150 µl of x1 MSD Read Buffer T and concentrations were read on a Sector 6000 reader. Concentrations were calculated using MSD Workbench software package (MesoScaleDiscovery, UK). In previous analyses, the % coefficient of variation for within-batch runs was <5% and between-batch runs was <11% (unpublished). Stability of the analyte had previously been determined with three serum samples split into four aliquots and stored either at −20°C, 4°C for 48 hours, room temperature for 48 hours or subjected to five freeze-thaw cycles. None caused a significant change in the measured FOLR1 concentration (unpublished). For quality control in the current investigation, three subject pools with low (319–506 pg/ml), mid (810–1,121 pg/ml) and high (1,446–2,108 pg/ml) ranges of serum FOLR1 concentration were analysed at the beginning and end of each assay. The average percent change was 5.2% for low range values, 14% for mid-range values and 2% for high-range values.

### Power and Statistical Analysis

We calculated 80% power to detect a difference in serum FOLR1 concentration of at least 23.9 pg/ml (assuming a standard deviation of 33 pg/ml) between groups using a two-sample t-test for independence and assuming alpha = 0.05 with 15 subjects for each gender comprising membership in extreme categories of risk factors (e.g., low [≤250 µg/d] vs high [≥600 µg/d] total folate intake). We based our calculation on the mean and standard deviation values of serum FOLR1 protein in controls in the study by Basal et al [29].

We applied non-parametric statistics to evaluate differences in untransformed serum FOLR1 concentrations between groups using Kruskal-Wallis tests. We also applied t-tests and ANOVA to serum FOLR1 transformed with the natural logarithm to improve normality. Since results from group comparisons were relatively similar using parametric and non-parametric tests, only the P values from the non-parametric tests are presented. To evaluate predictors of serum FOLR1 concentrations, we transformed serum FOLR1 with the natural logarithm and regressed values onto subject characteristics using general linear regression models, combining information from men and women, where possible, and adjusting for gender.

Statistical tests were two-sided and implemented with SAS version 9 (SAS Institute, NC) software. P values <0.05 were considered statistically significant and those between 0.05 and 0.10 suggestive of a trend association.

## Results

Serum FOLR1 concentration was higher in women (mean = 483 pg/ml, median = 491 pg/ml, range = 327–693 pg/ml) than in men (mean = 428 pg/ml, median = 404 pg/ml, range = 340–682 pg/ml) and this difference was statistically significant in univariable analysis (P = 0.001) (**Table 1**). Serum FOLR1 concentration was also positively associated with vitamin A intake (P = 0.02) and positive trend associations were observed with age and oral contraceptive use and an inverse trend association was observed with BMI (**Table 1**). There was no statistically significant difference in serum FOLR1 concentration between extreme categories of folate intake overall or for women or men separately, and no statistically significant differences across age categories when stratified by gender.

**Table 1:** Serum FOLR1 concentrations by participant characteristics.

Characteristic	N	Mean (SD) pg/ml	Median pg/ml	P value*
Gender				
Men	30	428 (76)	404	0.001
Women	30	483 (77)	491	
Age, years				
All subjects				
≤45	8	401 (61)	370	0.05
46–55	23	456 (91)	436	
>55	29	470 (73)	484	
Men				
≤45	6	377 (39)	366	0.13
46–55	14	449 (86)	432	
>55	10	428 (68)	402	
Women				
≤45	2	473 (67)	473	0.40
46–55	9	468 (102)	488	
>55	19	492 (67)	499	
Folate, µg/d				
All subjects				
≤250	30	445 (87)	413	0.29
≥600	30	465 (75)	474	
Men				
≤250	15	431 (92)	396	0.85
≥600	15	424 (59)	429	
Women				
≤250	15	459 (81)	499	0.38
≥600	15	507 (67)	489	
Vitamin A, µg/d				
All subjects				
≤2000	42	440 (80)	417	0.02
>2000	18	491 (74)	485	
Men				
≤2000	23	419 (80)	396	0.06
>2000	7	457 (56)	455	
Women				
≤2000	19	466 (73)	489	0.29
>2000	11	513 (79)	493	
BMI, kg/m2				
≤25	30	473 (78)	480	0.06
≥30	30	437 (81)	413	
Alcohol use				
No	5	477 (38)	480	0.38
Yes	55	453 (84)	438	
Oral contraceptive use†				
Never	15	460 (66)	481	0.05
Ever	15	507 (83)	504	
Hormone replacement use†				
No	19	478 (85)	488	0.38
Yes	11	492 (65)	499	
Breast feeding, months†				
0 months	7	517 (43)	511	0.38
1–12 months	9	444 (89)	487	
12–49 months	10	489 (86)	490	
Missing	4	–	–	
Pregnancies, any†				
1–2	14	492 (67)	496	0.34
3–10	12	468 (96)	484	
Missing	4	–	–	
Smoking				
Not at all	28	462 (75)	459	0.61
Occasionally/Daily	2	435 (69)	435	
Don’t know/missing	30	450 (89)	422	

*P values are from non-parametric statistics using the Kruskal-Wallis test.

† Among women only.

In a multivariable-adjusted linear regression model, inclusion of all variables in Table 1 explained 44% of the variation in serum FOLR1 protein (P = 0.04). However, only gender (P = 0.003) and vitamin A intake (P = 0.03) were statistically significant. A trend association was observed for oral contraceptive use among women (**Table 2**). The retention of gender and vitamin A intake in the regression model explained 20% (model *R^2^* = 0.20, P = 0.001) of the variation in serum FOLR1 concentration.

**Table 2:** Predictors* of serum FOLR1 concentrations.

Predictor	df†	Full Model	Reduced Models	
		P value	P value	P value
Age	2	0.09	–	–
Gender, female vs male	1	0.003	0.003	0.003
Vitamin A intake, >2000 vs ≤2000 µg/d	1	0.34	0.03	0.03
Oral contraceptive use, ever vs never	1	0.04	0.07	–
Folate intake	1	0.94	–	–
BMI	1	0.08	–	–
Breast feeding	2	0.26	–	–
Pregnancy	1	0.05	–	–
Smoking	2	0.37	–	–
Total energy, kcals/d	1	0.60	–	–
Alcohol	1	0.87	–	–
Hormone replacement use	1	0.80	–	–
Model R^2^		0.44	0.24	0.20
Model P value		0.04	0.001	0.001

*P values are from Type III sum of squares obtained from general linear regression models.

† degrees of freedom.

## Discussion

We evaluated various dietary, lifestyle, medical and reproductive factors with serum FOLR1 concentrations among healthy men and women and observed the strongest predictor to be female gender. Although we initially believed this finding to be novel, this occurrence was, in fact, first recognized 40 years ago, albeit when serum FOLR1 protein was first referred to as a serum folate binding protein [24], and we suspect that these are the same protein. Despite different median values for serum FOLR1 concentration between men and women, the range of normal values was similar (327–693 pg/ml).

Early studies of correlates of total serum folate binding capacity found positive associations with pregnancy [25], [26], oral contraceptive use [25]–[27] and female gender [27] but no association with folate nutritional status [25]. Similarly, in the current study, the difference in total folate intake was not a statistically significant predictor of serum FOLR1 concentrations. We observed a mean difference across folate intake categories of 48.0 pg/ml among women but only 7.0 pg/ml among men. Although we exceeded the minimum difference of 23.9 pg/ml that we calculated would provide 80% power to detect a statistical difference, the observed standard deviation was two-fold greater than the estimate we used in our power calculation [29]. This resulted in insufficient statistical power to assess whether a mean serum FOLR1 protein difference of 48.0 pg/ml between folate intake categories among women was statistically significant. Nevertheless, in multivariable linear regression, total folate intake was not a significant predictor of serum FOLR1 concentration. Our findings indirectly support the speculation that serum folate binders/FOLR1 protein may be influenced by female sex hormones [27]. Although we found no statistically significant association between number of pregnancies, months of breast-feeding or oral contraceptive use with serum FOLR1, these were not current events among women enrolled in our study as they were in previous reports [25]–[27]. However, our results suggest that past pregnancies, breast-feeding or oral contraceptive use may not have sustained influences on serum FOLR1 concentrations.

Membrane-bound FOLR1 has generated much interest as a diagnostic and chemotherapeutic target on the basis of observations of high expression in some cancers relative to healthy tissue [5]–[8]. As such, researchers have also examined whether serum FOLR1 could be a potential marker for early detection of female-related cancers, notably ovarian carcinoma [29]–[31]. The positive association between serum FOLR1 concentration and female gender independent of age suggests caution against statements to exploit serum FOLR1 for early cancer detection without further investigation into reasons for its presence in sera of women without cancer. One study found significantly higher serum FOLR1 levels among 15 early-stage ovarian carcinoma patients than among 30 healthy women using a microfiltration assay; however, the authors reported much lower overall serum FOLR1 concentrations among healthy women (mean: ∼0.7 nmol/L [SD = 0.1] or 231 pg/ml [SD = 33]) [29] than observed in the current study. That study also observed no association between age and serum FOLR1 concentrations, although the range of serum FOLR1 concentrations was much narrower [29] than in the current report. More recently, significant differences (P<0.0001) were observed for median serum FOLR1 concentrations between 100 cancer-free women (median = 500 pg/ml) and 100 patients with ovarian carcinoma overall (median = 1,500 pg/ml), and separately for early-stage (median = 1,100 pg/ml) and late-stage (median = 2,200 pg/ml) ovarian carcinoma, using an enzyme-linked immunosorbent assay [30]. In that study, the reported mean values were much lower among the cancer patients (mean = ∼900 pg/ml), and possibly even lower for early-stage cancers (data not reported), and the range of serum values overlapped considerably between normal and cancer groups [30]. Of note, the median serum FOLR1 concentration among controls in that study [30] was similar to that among women in the current investigation. These estimates provide investigators of future studies with mean and median values, standard deviations and range of normal values that are more accurate in order to plan studies with improved statistical power.

Several investigations provided evidence that membrane-bound FOLR1 expression was hormone mediated. Using HeLa and ovarian cell cultures and a luciferase assay, Kelley et al reported that the *FOLR1* gene promoter was repressed in the presence of 17β-estradiol and de-repressed by pharmacologic concentrations of the anti-estrogen tamoxifen in a dose-dependent manner [9]. In mouse embryonic stem cells, administration of 1 µM all trans retinoic acid (stated by the authors to be a proven dose for differentiation in this cell system) caused upregulation of *FOLR1* gene expression as early as 3 hours after administration [15]. The authors concluded that the rapid upregulation represented a direct effect of retinoic acid, and the mechanism may be from activation of a retinoic acid responsive element. Induction of *FOLR1* expression by retinoic acid and activators of retinoic acid receptor has also been reported by others [34], [35]. In the current study, we reasoned that similar associations might be observed with serum FOLR1 protein. Androgen conversion to 17β-estradiol in adipose tissue by the CYP19A1 (aromatase) enzyme is an important source of bioactive endogenous estrogens [36], particularly among postmenopausal women. The weak inverse association in univariable analyses between obesity assessed by BMI and serum FOLR1 concentrations indirectly supports the *in vitro* observations that the *FOLR1* gene promoter is repressed in the presence of 17β-estradiol [9]. Higher vitamin A (retinoic acid) intake >2,000 µg/d (approximately 3 times the recommended dietary intake for adults [37]) was associated with higher serum FOLR1 concentrations independent of age, gender and BMI and agrees with the observations from cell culture studies [15]. Retinoid responsive genes that are transcriptionally regulated contain consensus retinoic acid responsive elements within their promoter sequences. An early study identified a steroid receptor-binding element upstream of exon 4 in the alternate promoter P4 of the *FOLR1* gene [38], [39]. Conceivably, both female sex hormones and retinoic acid could regulate *FOLR1* gene expression. Retinoic acid is an essential vitamin important for normal vision, gene expression, reproduction, embryonic development, growth and immune function [37] and *FOLR1* gene expression may be implicated with one or more of these functions.

Limitations of the current investigation include the small sample size that did not permit exploration of subset analyses beyond those formally hypothesized. Furthermore, the broad questions eliciting information on oral contraceptive use and hormone replacement did not make it possible to evaluate the influence of different hormone preparations. The dietary data were collected several years prior to blood sampling and may not accurately reflect nutrient status at the time we evaluated serum FOLR1 protein concentrations. Although these associations should be interpreted cautiously, they appear to be consistent with earlier reports. Notable strengths, however, include the use of a sensitive clinical assay to measure serum FOLR1 protein and the analysis of sera from relatively healthy men and women representative of a general population.

In conclusion, we provide contemporary evidence to support the hypothesis from 40 years earlier that female gender and, by association, steroid hormones possibly influence serum FOLR1 concentrations. These results suggest additional larger studies among participants without cancer are required to confirm our findings, such as those from cohort studies with prospectively collected epidemiological data, in order to understand the biological underpinnings of the observed associations.
